# A retrospective study on the incidence, management and risk factors of skin rash in patients with advanced prostate cancer in Japan

**DOI:** 10.1186/s12894-023-01246-1

**Published:** 2023-04-28

**Authors:** Raf De Moor, Yosuke Koroki, David Bin-Chia Wu, Dae Young Yu, Mikiko Tohyama, Chikara Ohyama

**Affiliations:** 1 Integrated Market Access, Janssen Pharmaceutical K.K., 3 Chome-5-2 Nishikanda, Tokyo, 101-0065 Japan; 2 Medical Affairs, Janssen Pharmaceutical K.K., Tokyo, Japan; 3 Janssen Pharmaceutical Companies of Johnson and Johnson, Asia Pacific Regional Office, Singapore, Singapore; 4 Saw Swee Hock School of Public Health, National University of Singapore, Singapore, Singapore; 5 School of Pharmacy, Monash University Malaysia, Bandar Suwnay, Malaysia; 6 Department of Dermatology, National Hospital Organization Shikoku Cancer Center, Matsuyama, Japan; 7 Department of Urology, Hirosaki University Graduate School of Medicine, Hirosaki, Japan

**Keywords:** Japan, Prostate cancer, Real-world study, Skin rash

## Abstract

**Background:**

Worldwide, prostate cancer (PC) is the second most diagnosed cancer and the fifth leading cause of cancer death in men. Hormonal therapies, commonly used for PC, are associated with a range of treatment-emergent adverse events (TEAEs). The population from Japan seems to be at higher risk of developing TEAEs of skin rash compared to the overall global population. This study was conducted to get a better insight into the incidence, management, and risk factors for skin rash during active treatment for advanced PC in Japan.

**Methods:**

A retrospective cohort of PC patients was identified and subsequently categorized, into non-metastatic and metastatic castration-resistant prostate cancer patients (nmCRPC and mCRPC), and metastatic castration-naïve prostate cancer patients (mCNPC). The analysis was based on a dataset from the Medical Data Vision (MDV) database. Descriptive statistics were determined, and a multivariate Cox proportional hazards model was used to the associated risk factors for the onset of rash.

**Results:**

Overall, 1,738 nmCRPC patients, 630 mCRPC patients, and 454 mCNPC patients were included in this analysis. The median age was 78 years old and similar across the three cohorts. The skin rash incidence was 19.97% for nmCRPC cohort, 28.89% for mCRPC cohort, and 28.85% for mCNPC cohort. The median duration of skin rash ranged from 29 to 42 days. Statistically significant risk factors for developing skin rash included a history of allergy or hypersensitivity (all cohorts), increased age (nmCRPC and mCRPC), a body mass index (BMI) of < 18.5 (nmCRPC and mCRPC), and a PSA level higher than the median (nmCRPC). Skin rash was commonly managed with systemic and topical corticosteroids which ranged from 41.76% to 67.03% for all cohorts. Antihistamines were infrequently used.

**Conclusion:**

This study provides a better understanding of the real-world incidence, onset, duration, management and risk factors of skin rash in patients on active PC treatment in Japan. It was observed that approximately 20–30% of PC patients experience skin rash. Development of skin rash was associated with previous allergy or hypersensitivity, BMI of < 18.5, increased age and higher PSA levels, and was usually treated with corticosteroids.

**Supplementary Information:**

The online version contains supplementary material available at 10.1186/s12894-023-01246-1.

## Introduction

Prostate cancer (PC) is the second most frequent malignancy after lung cancer, accounting for approximately 1.27 million new cases annually and the fifth leading cause of death in men worldwide [1]. Although PC is more common in the western world and, the second most common cause of cancer-related mortality in the USA and Europe [2, 3], the proportion of patients with PC has been growing steadily in Japan which may be attributed to changes in lifestyle and diet [2, 4, 5]. The incidence of PC exceeded that of stomach and lung cancers, making PC the leading type of cancer in men from Japan in 2015 [5]. Advanced PC, including metastatic castration**-**resistant prostate cancer (mCRPC), non**-**metastatic castration**-**resistant prostate cancer (nmCRPC), and metastatic castration**-**naïve prostate cancer (mCNPC), is characterized by rising prostate**-**specific antigen (PSA) levels—indicating a significant risk for the development of disease progression and PC**-**specific death [6].

Hormonal therapies can delay the onset of metastasis and are the preferred treatment option for advanced PC, owing to their ease of administration and tolerability in many patients [7–9]. Systemic therapies using antiandrogens such as bicalutamide, abiraterone acetate (plus prednisolone), apalutamide, and enzalutamide have shown better survival benefits when administered along with androgen deprivation therapy (ADT) [10]. Although systemic therapies are highly efficacious, they are also associated with treatment-related adverse events (TEAEs) that may affect the patient’s quality of life [11].

Hormonal therapies, due to their interaction with various organs besides the prostate, are associated with a wide range of TEAEs such as loss of bone density, bone fractures, changes in blood lipids, insulin resistance, and weight gain. Skin rash has been observed in approximately 10% of patients with prostate cancer [12], can present itself in various forms, and is graded based on severity and body surface area (BSA) [12, 13]. The elderly population, irrespective of any underlying comorbidities seem to be at particular risk of developing skin conditions [14–16].

A real-world evidence study based on data from the US showed that, among patients with non-metastatic prostate cancer (nmPC), fatigue/asthenia (15.6%) and skin rash (10.9%) were the most common TEAEs associated with bicalutamide, abiraterone acetate (plus prednisolone), and enzalutamide treatments [12]. The high occurrence of skin rash has also been observed for patients in Japan after the administration of enzalutamide plus ADT (11.1%) [17], with apalutamide in the SPARTAN study and with apalutamide plus ADT in TITAN study. Skin rash was observed in 51.5% of the patients, and the incidence rates of rash 19.1%, generalized rash 16.2%, and maculo-papular rash 16.2% were also higher than that observed in the global study [13]. Of particular note for apalutamide, is the incidence of skin rash in the cohort from Japan being nearly double the incidence of the overall population of those studies. However, it is unclear whether a dermatologist had been involved in the diagnosis and if a definite causal relationship of the rash events with the PC medications had been determined. Hence, these cases of skin rash attributed to PC medications may have also included types of rash, which are common in the elderly [14] but are not drug-induced.

According to the SPARTAN study, the onset of skin rash during apalutamide administration was reported at a median of 82 days of treatment and resolved for the majority of patients within a median of 60 days [8]. Treatments to resolve skin rashes of less severity (Grades 1 and 2) included topical corticosteroids/oral antihistamines. Grade 3 skin rash was managed by systemic corticosteroids, dose modification or interruption [18].

The increased susceptibility to the development of rash in the Japanese population was previously noted following the launch of sodium-dependent glucose co-transporter type 2 (SGLT2) inhibitors in Japan in 2014 [19]. Only a few studies have evaluated the clinical profiles of patients that develop skin rash during the treatment of advanced PC, including nmCRPC, mCRPC, and mCNPC—particularly in real-world settings in Japan [20]. This real-world study was designed to obtain a better understanding of the incidence rate, management, and clinical characteristics of patients with skin rash during active treatment for advanced PC in Japan.

## Methods

### Study design

This retrospective cohort study was based on data extracted from the Medical Data Vision (MDV) database, a Japanese hospital-based claims database that contains standardized healthcare insurance claims data of more than 38 million individuals. This study was approved by the sponsor’s internal approval committee and in accordance with Japanese ethical and legal guidelines. The data is provided by hospitals using the Japanese Diagnosis and Procedure Combination fixed payment reimbursement system [21]. The considered time period ranged from April 2008 and January 2021.

### Patients

Patients with PC were included upon confirmed PC diagnosis according to the criteria outlined in the International Classification of Diseases 10th edition (ICD-10: C61) [22]. In addition, all patients had to have at least one surgical or medical castration (i.e. ADT) and one PSA measurement during the study period. Following inclusion, the PC cohort was further classified into nmCRPC, mCRPC, and mCNPC according to predefined inclusion criteria as outlined in Table 1.

**Table 1:** Inclusion criteria

nmCRPC	mCRPC	mCNPC patients
Consecutive increases in PSA levels, three times with a minimum one-week interval with the last PSA > 2 ng/mL while minimal initial PSA level of ≥ 1 ng/mL or last PSA level of > 2 ng/mL during first-line medical castration	Consecutive increases in PSA levels, three times with a minimum one-week interval with the last PSA level of > 2 ng/mL while minimal initial PSA level of ≥ 1 ng/mL, or during first-line medical castration with the last PSA level of > 2 ng/mL	Had a confirmed diagnosis of metastatic cancer on or after the confirmed diagnosis of the PC
Had no record of metastatic cancer on or before the index date	Any record of metastatic cancer on or before the index date	Had any record of first-line surgical or medical castration on or after the diagnosis of prostate cancer and metastasis diagnosis (i.e. neither medical nor surgical castrations before the diagnosis of metastasis)

*PC* Prostate cancer, *PSA* Prostate-specific antigen, *mCRPC* Metastatic castration-resistant prostate, *nmCRPC* Non-metastatic castration-resistant prostate cancer, *ng* nanogram, *mCNPC* Metastatic castration-naïve prostate cancer, *mL* milliliter

Of note is that the classification of the different cohorts was done based on clinical characteristics of patients that are recorded in MDV database [8, 21, 23, 24]. This is a necessary simplification compared to clinical practice in which a range of diagnostic methods can be used to diagnose, grade and stage prostate cancer. Patients diagnosed with any cancer other than prostate cancer on or before the first confirmed diagnosis of PC were excluded from the analysis.

The index date of the analysis was defined as the first date of any record of nmCRPC, mCRPC or mCNPC condition whereby all eligibility criteria were met. A washout period, defined as the period prior to the index date during which patients could not have a record of confirmed PC diagnosis was applied. The period was set to 90 days. This was considered to be sufficient to exclude prevalent cases of nmCRPC, mCRPC, and mCNPC, and was done to reduce associated bias of prevalent cases. Patients were followed up from the index date until the first occurrence of skin rash, or up to the end of record due to death, drop out, or the end of the study period, whichever occurred first.

Skin rash diagnosis in the database was identified by (1) the corresponding skin rash ICD-10 code (Additional file 1: Table S1), combined with (2) record of corresponding skin rash treatment prescribed in the same month of the confirmed diagnosis of skin rash (Additional file 1: Table S2). In addition to skin rash diagnosis, we identified patients with eosinophilia as some evidence suggests androgen receptor signaling inhibitors are reported to induce eosinophilia associated with development of skin rash [25]. Eosinophilia was identified based on the corresponding ICD-10 codes and provided in Additional file 1: Table S3. Previous events of allergy and hypersensitivity, up to one year before the index date, were identified according to ICD-10 codes (Additional file 1: Table S4) [26].

**Fig. 1:**
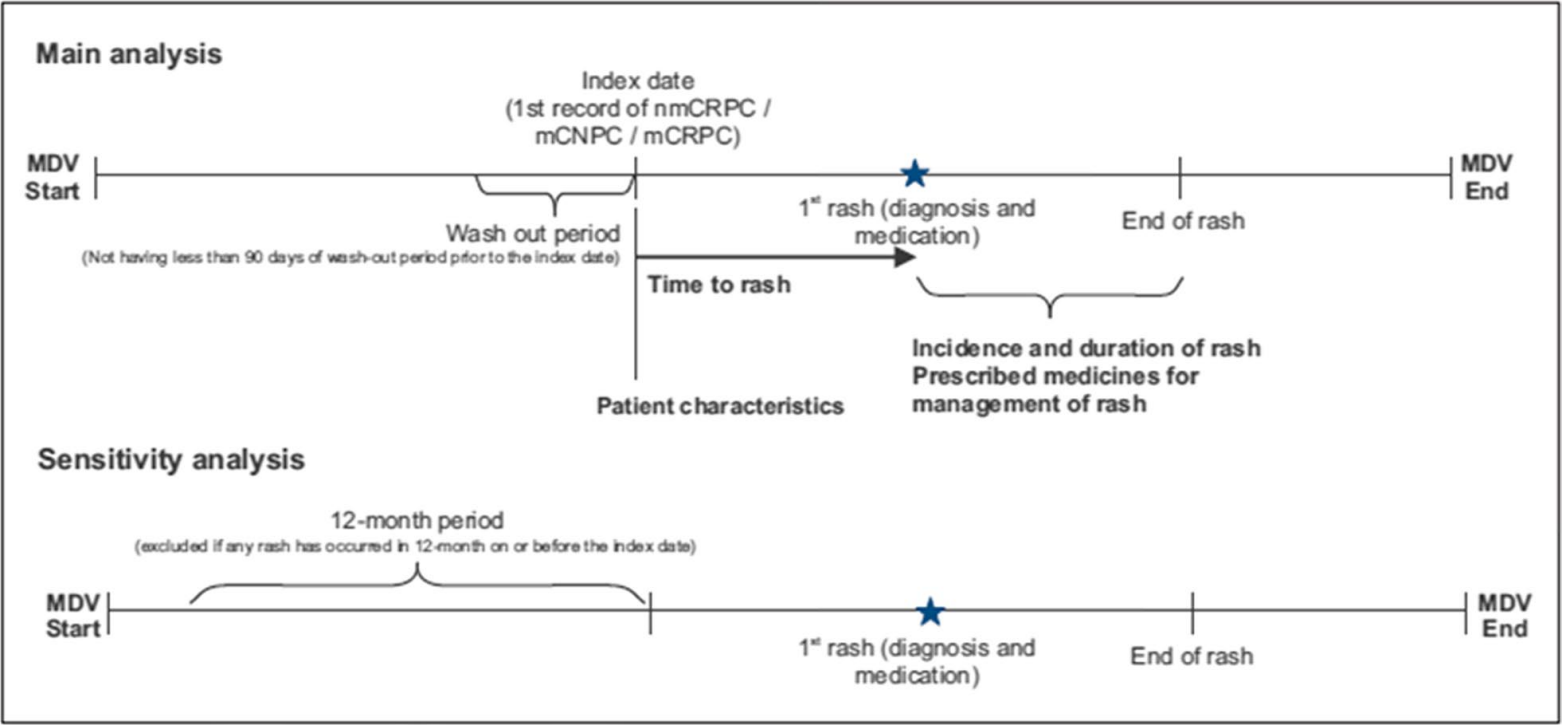
Study flow chart

A sensitivity analysis was conducted with an aim to exclude patients with chronic skin rash from the analysis. For this, patients who had any incidence of skin rash within 12 months prior to the index date were excluded from the dataset. This period was considered sufficient to identify and exclude any regular follow-up duration of chronic skin rash disease in Japan (Fig. 1). Missing data was not replaced by substitute values through imputation.

### Study variables

Patient baseline characteristics were determined at the index date and included age, height, weight, estimated glomerular filtration rate (eGFR), alanine aminotransferase (ALT), aspartate aminotransferase (AST), alkaline phosphatase (ALP), Gamma-glutamyltransferase (GGT), PSA value, and previous treatment for PC (e.g., radical prostatectomy, radiotherapy, and hormonal therapy) before the index date. For each cohort, descriptive statistics were calculated for the baseline characteristics, skin rash incidence, type of skin rash treatment and counts of prescribed medicines for the management of skin rash; and duration of skin rash treatments by administration route. Oral antihistamine, systemic corticosteroid, and topical corticosteroid were the administration routes considered to treat skin rash. For all administration routes, the last consecutive treatment was defined by either no diagnosis/rash treatment, or a treatment gap of more than 90 days from the end of the skin rash treatment designated for each drug formulation. The end of skin rash treatment for oral antihistamine was set to the end of the prescribed period. The prescribed period started at the date of the prescription and ended one day before the end of the prescribed period. For systemic corticosteroids, the prescription date was assumed to be the last day of the prescribed period. For topical corticosteroids, it was assumed a prescription would be provided for 28 days. This assumption was based on expert opinion. Similar to oral administration, the prescribed period ended one day before. The study outcomes assessed with their description are outlined in Table 2.

**Table ﻿2:** Study outcomes assessed

Variable	Description
Incidence proportion of skin rash	The proportion of new cases of skin rash during a specific period
Patient-year incidence rate	Period between the index date to the first diagnosis of skin rash and defined as the ratio of number of new cases of skin rash to the total time the population was at risk of skin rash
Types and counts of prescribed medications for management of skin rash	The type of drug (oral antihistamine, systemic corticosteroid, and topical corticosteroid) and number of patients receiving each type of skin rash treatment
Duration of skin rash	The time from initial record of skin rash treatment for patients diagnosed with skin rash to the last record of skin rash diagnosis and skin rash treatment with treatment durations
Drugs administered between index date and the first onset of skin rash	The number and proportion of patients receiving prostate cancer treatment between the index date and the first onset of skin rash

A multivariate Cox proportional hazards model analysis was done to determine risk factors associated with the development of skin rash. The Cox proportional hazard model [27] can be expressed as:

$$\mathrm{h}\left(\mathrm{t}\right)=\mathrm{h}\left(\mathrm{t}|{\mathrm{x}}_{1}, {\mathrm{x}}_{2},\dots ,{\mathrm{x}}_{\mathrm{p}}\right)={\mathrm{h}}_{0}\left(\mathrm{t}\right)\mathrm{exp}({\upbeta }_{1}{\mathrm{x}}_{1}+{\upbeta }_{2}{\mathrm{x}}_{2}+\dots +{\upbeta }_{\mathrm{p}}{\mathrm{x}}_{\mathrm{p}}),$$

where ${\upbeta }_{1}, {\upbeta }_{2},\dots ,{\upbeta }_{\mathrm{p}}$ are unknown regression coefficients that are associated with ${\mathrm{x}}_{1}, {\mathrm{x}}_{2},\dots ,{\mathrm{x}}_{\mathrm{p}}$ and ${\mathrm{h}}_{0}\left(\mathrm{t}\right)$ is an unspecified baseline hazard function. The survival function can be linked to $\mathrm{h}\left(\mathrm{t}\right)$ and expressed as

$$\mathrm{S}\left(\mathrm{t}\right)=\mathrm{S}\left(\mathrm{t}|{\mathrm{x}}_{1}, {\mathrm{x}}_{2},\dots ,{\mathrm{x}}_{\mathrm{p}}\right)={\left[{\mathrm{S}}_{0}\left(\mathrm{t}\right)\right]}^{\mathrm{exp}({\upbeta }_{1}{\mathrm{x}}_{1}+{\upbeta }_{2}{\mathrm{x}}_{2}+\dots +{\upbeta }_{\mathrm{p}}{\mathrm{x}}_{\mathrm{p}})}$$

where ${\mathrm{S}}_{0}\left(\mathrm{t}\right)=\mathrm{exp}\left(-{\int }_{0}^{\mathrm{t}}{\mathrm{h}}_{0}\left(\mathrm{u}\right)\mathrm{du}\right)$ is the baseline survival function.

## Results

### Patient disposition and characteristics

**Fig. 2:**
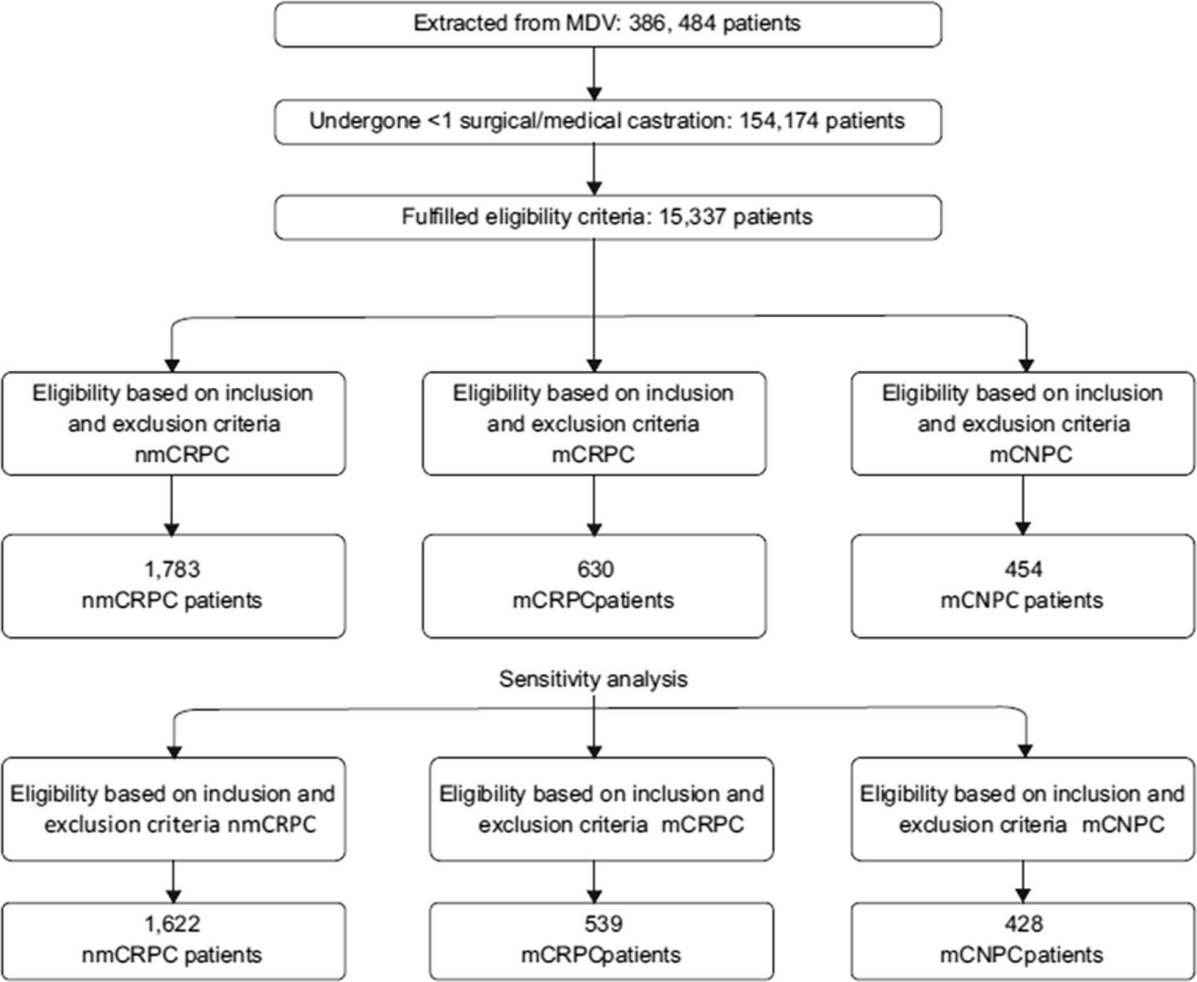
Flow diagram of patient eligibility. *MDV* Medical Data Vision, *mCNPC* Metastatic castration-naïve prostate cancer, *mCRPC* Metastatic castration-resistant prostate, *nmCRPC* Non-metastatic castration-resistant prostate cancer

An overview of the patient flow is provided in Fig. 2. Overall, a total of 386,484 patients were identified with confirmed PC. Of this cohort, 154,174 patients were found to have undergone at least one surgical or medical castration and 15,337 patients had a PSA measurement during the study period.

After applying the inclusion and exclusion criteria for each cohort, as outlined in (Fig. 2), patients were categorized as 1783 patients for nmCRPC, 630 patients for mCRPC, and 454 patients for mCNPC for the overall analysis (Table 3).

**Table 3:** Attrition

Overall population		
	Patients excluded N (%)	Patients remaining N (%)
Inclusion criteria		
1. Diagnosis of prostate cancer without a suspicious flag	0 (0.0%)	386,484 (100.0%)
2. Having at least one surgical or medical ADT during the study period	232,310 (60.1%)	154,174 (39.9%)
Exclusion criteria		
1. Not having a PSA measurement during the study period	138,837 (87.9%)	15,337 (12.1%)
*Cohort (nmCRPC)*		
Inclusion criteria		
1. Consecutive increases in PSA levels, 3 times with at least one-week interval with the last PSA > 2 ng/mL while minimal staring PSA level of ≥ 1 ng/mL, or during first-line medical castration with the last PSA > 2 ng/mL	10,749 (70.0%)	4588 (30.0%)
2. Having any record of the first-line surgical or medical castration on or before the index date	528 (12.9%)	4060 (87.1%)
3. Having no record of metastatic cancer on or before index date	1506 (40.0%)	2554 (60.0%)
Exclusion criteria		
1. Diagnosis of any cancer other than prostate cancer on or before first confirmed diagnosis of prostate cancer	256 (9.5%)	2298 (90.5%)
2. Having less than 90 days of washout before the index date	515 (22.4%)	1783 (77.6%)
*Cohort (mCRPC)*		
Inclusion criteria		
1. Consecutive increases in PSA levels, 3 times with at least one-week interval with the last PSA > 2 ng/mL while minimal initial PSA level of ≥ 1 ng/mL, or during first-line medical castration with the last PSA > 2 ng/mL	10,749 (70.0%)	4588 (30.0%)
2. Having any record of the first-line surgical or medical castration on or before the index date	528 (12.9%)	4060 (87.1%)
3. Any record of metastatic cancer on or before the index date	2554 (60.0%)	1506 (40.0%)
Exclusion criteria		
1. Diagnosis of any cancer other than prostate cancer on or before the first confirmed diagnosis of prostate cancer	793 (47.0%)	713 (53.0%)
2. Having less than 90 days of washout before the index date	83 (11.6%)	630 (88.4%)
*Cohort (*mCNPC*)*		
Inclusion criteria		
1. Diagnosis of metastatic cancer on or after the diagnosis of the prostate cancer	11,617 (76.8%)	3720 (23.2%)
2. Any record of the first-line surgical or medical castration on or after the diagnosis of the prostate cancer and the diagnosis of the metastasis	1599 (39.6%)	2121 (60.4%)
Exclusion criteria		
1. Diagnosis of any cancer other than prostate cancer on or before the first confirmed diagnosis of prostate cancer	143 (8.7%)	1978 (91.3%)
2. Having less than 90 days of washout before the index date	1524 (77.0%)	454 (23.0%)

*ADT* Androgen deprivation therapy, *mCNPC* Metastatic castration-naïve prostate cancer, *mCRPC* Metastatic castration-resistant prostate cancer, *N* number of patients in a specific group, *nmCRPC* Non-metastatic castration-resistant prostate cancer, *PSA* Prostate-specific antigen

**Fig. 3:**
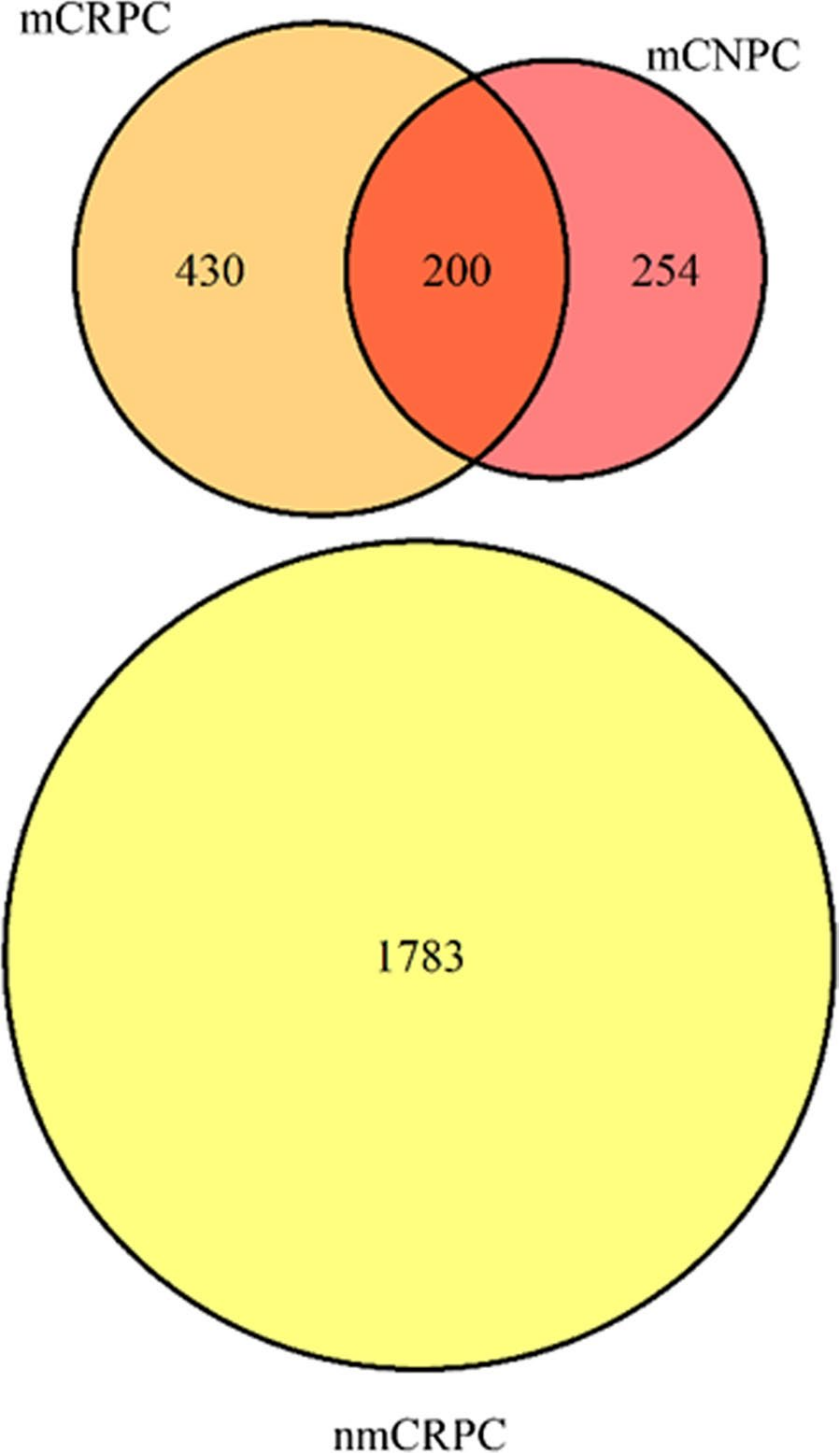
Venn diagram of study population

A section of patients overlapped between mCRPC and mCNPC, when patients were initially identified as mCNPC but progressed to mCRPC (Fig. 3). For the sensitivity analysis (Fig. 2), a total of 1,622 patients, were included for nmCRPC, 539 patients, for mCRPC, and 428 patients for mCNPC cohorts.

### Baseline characteristics

Baseline characteristics of the population (Table 4) were determined at the index date.

**Table 4:** Patient characteristics

	Overall	nmCRPC	mCRPC	mCNPC
	N = 2667	N = 1783	N = 630	N = 454
*Age on the index date (years)*				
Mean (SD)	77.5 (8.08)	78.3 (7.99)	76.3 (7.92)	76.2 (8.40)
Median	78	79	76	76
IQR (Q1:Q3)	11 (72–83)	11 (73–84)	11 (71–82)	12 (70–82)
*Age group at the index date, n (%)*				
18–49	1 (0.04%)	0 (0.00%)	1(0.16%)	0 (0.00%)
50–59	39 (1.46%)	23 (1.29%)	11 (1.75%)	10 (2.20%)
60–69	410 (15.37%)	238 (13.35%)	112 (17.78%)	87 (19.16%)
70–79	1075 (40.31%)	683 (38.31%)	283 (44.92%)	189 (41.63%)
80–89	994 (37.27%)	724 (40.61%)	200 (31.75%)	145 (31.94%)
90+	148 (5.55%)	115 (6.45%)	23 (3.65%)	23 (5.07%)
*Height (centimeter)*				
Mean (SD)	162.3 (7)	162.3 (6.72)	162.2 (8.01)	162.1 (7.85)
Median	162	161	161	162
IQR (Q1:Q3)	10 (156–166)	10 (156–166)	11 (155–166)	9 (157–166)
Missing	710	548	129	63
*Weight (kilogram)*				
Mean (SD)	61.3 (11.00)	61.7 (10.98)	60.3 (11.60)	60.5 (11.47)
Median	59.80	60.00	59.05	59.20
IQR (Q1:Q3)	15.42 (51.58–67.00)	15.37 (51.90–67.27)	15.75 (50.00–65.75)	15.02 (51.98–67.00)
Missing	696	539	124	62
*eGFR (mL/min/1.73 m*^*2*^*)*				
Mean (SD)	64.0 (21.53)	63.1 (20.19)	67.2 (25.27)	63.7 (23.98)
Median	64.38	63.52	67.61	64.70
IQR (Q1:Q3)	25.29 (50.88–76.17)	24.79 (50.42–75.21)	27.91 (54.00–81.91)	25.29 (50.64–75.93)
Missing	18	17	0	1
*ALT (U/L)*				
Mean (SD)	22.2 (22.97)	22.8 (24.26)	21.1 (22.22)	20.1 (14.41)
Median	17.0	18.0	16.0	16.5
IQR (Q1:Q3)	12 (13.00–25.00)	13 (13.00–26.00)	10.25 (12.00–12.25)	10 (13.00–23.00)
Missing	77	60	12	7
*AST (U/L)*				
Mean (SD)	27.4 (24.07)	27.0 (21.88)	28.8 (34.16)	27.5 (16.72)
Median	23.0	23.0	23.0	23.5
IQR (Q1:Q3)	(19–29)	(19–29)	(18–29)	(19–30)
Missing	29	15	8	6
*ALP (U/L)*				
Mean (SD)	285.7 (596.58)	201.5 (201.33)	405.9 (660.83)	557.5 (1236.11)
Median	175.0	169.0	188.0	231.8
IQR (Q1:Q3)	96.5 (138.5–235.0)	76.0 (134.5–210.5)	153.5 (142.0–295.5)	332.76 (154.12–486.88)
Missing	150	97	23	40
*GGT(U/L)*				
Mean (SD)	40.0 (59.11)	37.9 (52.22)	43.5 (77.55)	48.3 (79.64)
Median	26	26	25	30
IQR (Q1:Q3)	24 (17.0–41.0)	23 (17.0–40.0)	11 (18.0–29.0)	24.75 (20.0–44.8)
Missing	881	620	208	120
*Previous treatment for prostate cancer, n (%)*				
Any RP	64 (2.40%)	45 (2.52%)	13 (2.06%)	8 (1.76%)
Any RT	201 (7.54%)	111 (6.23%)	80 (12.70%)	27 (5.95%)
Any hormonal therapy	1979 (74.20%)	1419 (79.58%)	550 (87.30%)	157 (34.58%)
*Baseline PSA at the index date (ng/mL)*				
N	2501	1783	630	288
Mean (SD)	111.50 (606.15)	29.52 (272.21)	144.95 (545.85)	629.28 (1429.08)
Median (Min–Max)	5.55 (0–12, 676.31)	4.52 (2.01, 8519.69)	12.15 (2.01, 9,070.83)	100.2 (0, 12, 676.31)
IQR(Q1-Q3)	15.64 (2.99, 18.63)	7.16 (2.82, 9.98)	55.31 (4.40, 59.71)	552.44 (14.66, 567.10)
Missing	166	0	0	166

*N* Number of patients in a specific group, *n* Number of patients without missing value in a specific group, *ALP* alkaline phosphatase, *ALT* alanine aminotransferase, *AST* aspartate aminotransferase, *eGFR* estimated glomerular filtration rate, *GGT* Gamma-glutamyltransferase, *IQR* Interquartile range, *L* liter, *Max* Maximum, *mCNPC* Metastatic castration-naïve prostate cancer, *mCRPC* Metastatic castration-resistant prostate cancer, *min* minutes, *mL* milliliter, *Min* Minimum, *ng* nanogram, *nmCRPC* Non-metastatic castration-resistant prostate cancer, *U* Units, *PSA* Prostate-specific antigen, *RP* Radical prostatectomy, *RT* Radiotherapy, *SD* Standard deviation

The mean values for age, and weight, renal function, and liver function were similar across all cohorts. Due to outliers, the median has been reported for PSA. Note that baseline PSA data was not available for 36.6% of patients in the mCNPC cohort. The majority of patients (74.2%) across all indications had received prior hormonal therapy, although this varied considerably across the cohorts and was 87.3% for mCRPC, 79.6% for nmCRPC, and 34.6% for mCNPC. Previous treatment with radical prostatectomy and radiotherapy was administered to less than 10% of the overall population. Baseline characteristics of the overall analysis were similar to those of the sensitivity analysis (Additional file 1: Table S5).

### Incidence of skin rash, duration and administered treatments

Approximately 29% of patients with mCRPC and mCNPC experienced skin rash. This incidence was notably higher compared to patients with nmCRPC (19.97%). Similarly, the incidence rate per 100 patient-years was highest in the mCRPC cohort (28.89%), followed by the mCNPC (28.85%) and nmCRPC cohorts (19.97%) (Table 5).

**Table 5:** Incidence and duration of skin rash, and administered treatments

	nmCRPC	mCRPC	mCNPC
	N = 1783	N = 630	N = 454
*Incidence proportion of skin rash (n, %)*^1^	356 (19.97%)	182 (28.89%)	131 (28.85%)
*Incidence rate of skin rash (per patient year)*^1^			
Patient years at risk (patient years)	3614.53	837.02	804.78
Incidence rate (per 100 patient years)	9.85	21.74	16.28
95% CI (lower–upper)	8.88–10.93	18.80–25.14	13.72–19.32
*Duration of skin rash (days)*			
n	356	182	131
Mean (SD)	138.30 (222.11)	120.50 (175.83)	134.10 (249.52)
Median	42 (1:1401)	42 (1:1196)	29 (1:1881)
IQR (Q1:Q3)	127 (28,155)	119 (28,147)	103 (28:131)
Missing	1427	448	323
*Drugs used for treatment of prostate cancer between index date (inclusive) and the first onset of skin rash (exclusive) (n, %)*^2^			
n	356	182	131
Goserelin	112 (31.46%)	44 (24.18%)	24 (18.32%)
Median duration (days)	591	459	590
Leuprorelin acetate	156 (43.82%)	57 (31.32%)	37 (28.24%)
Median duration (days)	468	336	430
Degarelix	36 (10.11%)	42 (23.08%)	43 (32.82%)
Median duration (days)	149	251	239
Flutamide	70 (19.66%)	32 (17.58%)	20 (15.27%)
Median duration (days)	142	125	81
Bicalutamide	148 (41.57%)	50 (27.47%)	84 (64.12%)
Median duration (days)	340	151	322
Enzalutamide	72 (20.22%)	32 (17.58%)	10 (7.63%)
Median duration (days)	295	237	275
Apalutamide	12 (3.37%)	2 (1.10%)	2 (1.53%)
Median duration (days)	112	93	279
Abiraterone Acetate (plus prednisolone)	38 (10.67%)	28 (15.38%)	17 (12.98%)
Median duration (days)	146	139	171
Darolutamide	0 (0.00%)	1 (0.55%)	0 (0.00%)
Median duration (days)	150	237	237
Docetaxel	62 (17.42%)	56 (30.77%)	16 (12.21%)
Median duration (days)	177	153	164
Cabazitaxel	14 (3.93%)	19 (10.44%)	3 (2.29%)
Median duration (days)	120	84	62
Radium-223	0 (0.00%)	5 (2.75%)	1 (0.76%)
Median duration (days)	0	139	139

*N* number of patients in a specific group, *n* number of patients occurred skin rash in a specific group, *CI* Confidence interval, *IQR* Interquartile range, *Max* Maximum, *mCNPC* Metastatic castration-naïve prostate cancer, *mCRPC* Metastatic castration-resistant prostate cancer, *Min* Minimum, *nmCRPC* Non-metastatic castration-resistant prostate cancer, *Q1* first quartile, *Q3* third quartile, *SD* Standard deviation

^1^Estimated by exact Poisson confidence interval

^2^Percentage among the number of patients with skin rash

The sensitivity analysis showed a slightly lower proportion of skin rash incidence (Additional file 1: Table S6). Similar to the reporting for PSA values, the median has been reported for the duration of skin rash due to outliers and it was shortest for the mCNPC cohort (29.0 days), followed by the nmCRPC and mCRPC cohorts (42.0 days for both). The sensitivity analysis showed a shorter duration of rash compared to the overall analysis, which was 28.0 days in the mCNPC cohort, 34.0 days in the mCRPC cohort, 35.5 days in the nmCRPC.

The most frequently administered treatment between the index date and the first onset of skin rash in the nmCRPC cohort was leuprorelin acetate (43.8%), followed by bicalutamide (41.6%) and goserelin (31.5%). Similar to nmCRPC, for the mCRPC cohort, leuprorelin acetate was the most common administered drug (31.3%), followed by docetaxel (30.8%), and bicalutamide (27.5%). For the mCNPC cohort, bicalutamide was predominantly administered (64.1%), followed by degarelix (32.8%) and leuprorelin acetate (28.2%).An overview of the administered treatments and the corresponding median treatment duration is provided in Table 5. In total 3 patients (0.1%) in the entire cohort were diagnosed with eosinophilia.

### Risk factors

Cox regression analysis was conducted to determine risk factors that are potentially linked with the onset of skin rash. A history of allergy and hypersensitivity up to one year before the index date was a statistically significant risk factors for all cohorts. For nmCRPC and mCRPC patients, increased age (90+ years), and BMI < 18.5 were statistically significant risk factors. For nmCRPC, an age between 80 and 89 years old and a PSA value higher than the median were also statistically significant risk factors.

A detailed overview of all risk factors, the corresponding hazard ratio (HR), confidence interval (CI), and p-value for each cohort is provided in Table 6. Similar results were obtained for the sensitivity analysis and are provided in (Additional file 1: Table S7).

**Table 6:** Cox regression analysis for time to skin rash

	nmCRPC				mCRPC				mCNPC			
	HR	95% CI (Lower)	95% CI (Upper)	p-value	HR	95% CI (Lower)	95% CI (Upper)	p-value	HR	95% CI (Lower)	95% CI (Upper)	p-value
*Age group at the index date*												
18–49	–				1.36	0.16	11.48	0.7760	–			
50–59	1			Ref	1			Ref	1			Ref
60–69	1.71	1.03	2.84	0.0395	1.17	0.50	2.76	0.7211	0.94	0.40	2.20	0.8805
70–79	2.00	1.22	3.29	0.0062	1.54	0.66	3.57	0.3162	0.97	0.42	2.21	0.9338
80–89	2.69	1.63	4.42	< 0.0001	1.93	0.83	4.48	0.1273	1.35	0.59	3.09	0.4747
90+	3.68	2.16	6.25	< 0.0001	3.83	1.49	9.82	0.0052	2.48	1.00	6.18	0.0508
*Baseline PSA*												
Median PSA	1.29	1.03	1.62	0.028	1.24	0.92	1.69	0.163	1.37	0.88	2.14	0.163
*BMI*												
BMI < 18.5	0.50	0.38	0.66	< 0.0001	0.49	0.33	0.74	0.0006	0.67	0.41	1.10	0.1130
18.5 ≤ BMI < 25	Ref											
BMI ≥ 25	0.89	0.68	1.16	0.3830	1.01	0.70	1.46	0.9569	0.97	0.64	1.47	0.8730
*Allergy and hypersensitivity*	1.89	1.37	2.59	< 0.0001	2.26	1.51	3.40	< 0.0001	1.84	1.09	3.12	0.023

In the Cox regression for PSA, < median PsA was considered as reference; in the Cox regression for BMI, a normal weight (18.5 ≤ BMI < 25) was considered as reference; in the Cox regression for allergy and hypersensitivity, non-history (no allergy or hypersensitivity) was considered as reference

*BMI* Body mass index, *CI* Confidence interval, *HR* Hazard ratio, *mCNPC* Metastatic castration-naïve prostate cancer, *mCRPC* Metastatic castration-resistant prostate cancer, *nmCRPC* Non-metastatic castration-resistant prostate cancer, *PSA* Prostate-specific antigen, *Ref* Reference level, *RP* Radical prostatectomy, *RT* Radiotherapy

### Management of skin rash

Corticosteroids were the most frequently prescribed treatment in all cohorts. Topical corticosteroids were the most frequently administered treatment for nmCRPC (59.27%) and mCNPC (59.54%) patients, while for mCRPC systemic corticosteroids were the most frequently prescribed treatment (67.03%). The use of oral antihistamines ranged from approximately 23% for mCRPC to 31% for mCNPC. The median has been reported for skin rash treatment duration due to the outliers and the subsequent impact on the mean. Overall, the median treatment duration was higher for systemic corticosteroids (51.5–63.5 days) than oral antihistamines (28.0 to 40.0 days), Table 7.

**Table 7:** Summary of prescribed medicines for management of skin rash

	nmCRPC	mCRPC	mCNPC
	N = 356	N = 182	N = 131
Types of skin rash treatment, n (%)			
Oral antihistamine^1^	104 (29.21)	42 (23.08%)	41(31.30%)
Systemic corticosteroid^1^	184 (51.69%)	122 (67.03%)	64 (48.85%)
Topical corticosteroid	211 (59.27%)	76 (41.76%)	78 (59.54%)
Duration of skin rash treatment (Day)			
Oral antihistamine^1^	104	42	41
Mean (SD)	101.29 (180.55)	101.12 (153.46)	158.37 (222.60)
Median (Min:Max)	28 (1:1169)	30 (1:613)	40 (1:872)
IQR (Min:Max)	98.5 (8:106.5)	100 (12:112)	167 (14:181)
Systemic corticosteroid^1^	184	122	64
Mean (SD)	167.42 (244.89)	119.56 (159.30)	141.05 (242.61)
Median (Min:Max)	63.5 (1:1430)	59.5 (1:753)	51.5 (1:1333)
IQR (Min:Max)	189 (21:210)	129 (24:153)	131.5 (19:150.5)
Topical corticosteroid	211	76	78
Mean	81.12 (149.55)	85.14 (159.90)	82.22 (160.95)
Median	28 (28:1350)	28 (28:1223)	28 (28: 1,131)
IQR	42 (28:70)	48.5 (28:76.5)	8 (28:36)

*N* number of patients in a skin rash specific group, *n* Number of patients without missing value in a specific group, *IQR* Interquartile range, *Max* Maximum, *mCNPC* Metastatic castration-naïve prostate cancer, *mCRPC* Metastatic castration-resistant prostate cancer, *Min* Minimum, *nmCRPC* Non-metastatic castration-resistant prostate cancer, *SD* Standard deviation

^1^Including combination of systemic antihistamine and corticosteroid

For topical corticosteroids the median treatment duration was equal to the assumption of the prescription period (28.0 days). Sensitivity analysis showed similar results for topical corticosteroids in the nmCRPC, mCRPC, and mCNPC cohorts, respectively (Additional file 1: Table S8).

## Discussion

In this retrospective cohort study, data from the MDV health claims database from Japan were analyzed for a 13 year-period. The objective was to gain better insight into understanding the baseline characteristics of patients that experience skin rash during PC treatment, as well as the incidence, duration, management, and risk factors for developing skin rash in the real-world setting.

Incidence of skin rash was noted in nearly 20–30% of the PC patients and was greater in metastatic (mCRPC [28.89%] and mCNPC [28.85%]) patients than in non-metastatic (nmCRPC) patients [19.97%]. Integrated analysis of the SPARTAN and TITAN study of Japanese patients showed that for 14.7% grade 3 skin rash was observed. Note that the usefulness of the comparison against All Grade from the integrated analysis of the SPARTAN and TITAN study is likely limited because the grade of skin rash cannot be identified from the database analysis and since it is likely that lower grades of skin rash are not reported with ICD-10 codes. A prior event of allergy or hypersensitivity, BMI < 18.5, higher age and higher baseline PSA levels were identified as statistically significant risk factors for development of skin rash that was most frequently managed with corticosteroids. The skin rash resolved comparatively sooner in the mCNPC cohort than in the nmCRPC and mCRPC cohorts. The incidence of PC has been increasing worldwide in recent years [28]. Although the treatment of PC using next generation antiandrogens such as apalutamide, enzalutamide, and darolutamide leads to more successful disease management, fatigue, diarrhea, and skin rash (> Grade 3) were observed as TEAEs of clinical significance with these drugs [12]. Evidence from randomized controlled studies, database studies, and pharmacovigilance demonstrated that skin rash may be associated with the administration of PC treatment [8, 12, 29, 30]. It should be noted that skin rash may be used as an umbrella term and can include different types such as butterfly rash, erythematous rash, exfoliative rash, follicular rash, generalized rash, macular rash, maculopapular rash, papules, papular rash, pruritic rash, pustular rash, genital rash, blister, skin exfoliation, exfoliative dermatitis, skin reaction, systemic lupus erythematosus rash, toxic skin eruption, mouth ulceration, drug eruption, conjunctivitis, erythema multiforme, stomatitis, and urticaria. Although skin rash does not have a significant impact on healthcare costs, it can have an impact on the overall quality of life of patients.

Incidence of skin rash was greater in metastatic (mCRPC and mCNPC) patients than in non-metastatic (nmCRPC) patients. The small difference in patient numbers between the overall analysis and sensitivity analyses implies that most eligible patients for the overall analysis did not have chronic skin rash.

The average age of the cohort was high, with a mean age of 77.5 years, whereby most patients had received prior hormonal therapy. Both values are in line with clinical expectations and are consistent with another integrated analysis in patients from Japan [13, 30]. The proportion of mCNPC patients receiving prior hormonal therapy (34.6%) at baseline is justified because patients with de novo metastatic prostate cancer and patients with localized prostate cancer who experienced disease recurrence after treatment with local therapy, including neo/adjuvant hormonal therapy, and diagnosed with metastatic PC are considered as patients with mCNPC. Also of note is that previous hormonal therapy (including surgical and medical castrations) for PC was measured in a period between the months of first confirmed diagnosis of prostate cancer (inclusive) to the month of first metastasis diagnosis (inclusive) in mCNPC cohort. Therefore, a mCNPC patient who received hormonal therapy in the same month of the first metastasis diagnosis was counted as one who underwent previous hormonal therapy.

The results showed that oral antihistamine, systemic and topical corticosteroids were used to treat skin rash in patients with advanced PC. In clinical practice, management of skin lesions due to apalutamide, antihistamines and topical steroids have been used primarily. Unfortunately, no literature was found about the management of skin rashes due to androgen receptor-signaling inhibitors. Leuprorelin acetate and bicalutamide were the most commonly prescribed drugs across all cohorts. It should be noted that apalutamide for mCRPC is not off-label in Japan and can be administered some urologists for this patient population. While on active PC treatment, approximately 20–30% of patients experienced skin rash. The corresponding incidence rate per 100 patient years ranged from 9.85 to 21.74.

The median duration of the skin rash ranged from 29 to 42 days. However, these values should be considered in light of the associated mean that was considerably higher than the median due to outliers. Hence, the actual duration of the skin rash could be longer. Furthermore, to determine the duration of skin rash for systemic corticosteroids and topical steroids in this database analysis, it was necessary to rely on assumptions pertaining to the prescription period which were based on expert opinion. The actual prescribed period in the claims database is only registered for oral drugs.

Considering that a previous event of allergy or hypersensitivity (HR = 1.09–1.89) is a significant risk factor for all cohorts suggests that the overreaction of the immune system is not tied to one cause or exposure, and that, once an overreaction of the immune system did occur, it is more likely to occur in the future. Underweight (BMI ≤ 18.5) was associated with a reduced chance to develop skin rash (HR nmCRPC = 0.50; HR mCRPC = 0.49). Overweight (BMI ≥ 25) was not linked with an increased risk to develop skin rash. It is no surprise that age is a significant factor as all skin layers alter over time which in turn increases the susceptibility to a wide range of skin problems [16]. Following the result on nmCRPC patients for which a PSA value higher than the median (4.52 ng/mL) is associated with a higher chance of developing skin rash (HR = 1.29), a pragmatic literature search was conducted to assess whether this relationship has been documented before. No prior publications or hypothesis were found on the correlation between high PSA levels and possible skin conditions. In addition to this, we also explored the correlation between age and PSA as advanced prostate cancer commonly occurs in elderly patients, and because an age-associated increase of PSA has been reported [31]. However, no correlation was found in any of the cohorts between age and PSA (Additional file 1: Table S9).

In this study, systemic corticosteroids were most frequently administered to treat skin rash among patients with mCRPC (67.0%), while topical corticosteroids were most commonly administered for patients with nmCRPC and mCNPC (~ 60% in both). Oral antihistamines were prescribed to nearly 30% of patients with skin rash. In the Uemura et al. [13] study, for patient who developed rash, almost half of them were treated with antihistamine, and > 50% of patients with rash were treated with topical corticosteroid. The higher severity of skin rash in this study, advocated by the higher rates of systemic and topical corticosteroids, may be due to the use of ICD-10 codes to identify skin rash as clinicians do not record diseases without treatment in routine practice. Additionally, use of systemic corticosteroids for conditions other than skin rash, even after excluding prednisolone in combination with abiraterone acetate and systemic corticosteroids in combination with docetaxel or cabazitaxel, may contribute to the difference between this study and the analysis of Uemura et al. Furthermore, the use of codes to identify rash and the uncertainty regarding the indication for corticosteroids may have limited the ability to accurately estimate the incidence of rash.

The median treatment duration of skin rash management was similar to the duration of skin rash and ranged between 28 and 63.5 days, which is in accordance with what has been observed in the SPARTAN study data [8]. In the global population of the SPARTAN study, skin rash of any grade resolved in 80.6% of patients within 60 days, while the median time to resolution of skin rash of any grade in the TITAN study was 100 days [8, 30]. In a retrospective pooled analysis, the median time to resolution of skin rash was 30 days [13]. The resolution of skin rash was faster among patients from Japan than in the SPARTAN study [8].

As this study is based on standardized health care insurance claims data with a representative geographic distribution across Japan, it is reflective of the overall population of Japan. Additionally, this study also provides details on the usage of anticancer drugs indicated for advanced PC treatment. The available study data from the MDV database was extensive, thus providing specific case identification and study measures for the patient subgroups. The results of this real-world evidence study are comparable to those noted in previous clinical studies [8, 30].

Limitations were related to those inherent to database analysis, including the lack of information on the severity of rash, inability to draw a causal relationship between PC treatment and rash, and limited clinical information to measure prostate cancer progression like pain progression and worsening of disease-related symptoms as assessed in the European Medicines Agency (EMA) [24] or SPARTAN trial [8]. Furthermore, the occurrence of rash likely depends on the season due to changes in humidity, temperature and personal hygiene. The impact of these differences due to seasonal changes and subsequent occurrence of skin rash could not be picked up in this study. It is noteworthy that various underlying reasons such as patient characteristics of age, weight, and disease progression may also play a role in the onset of skin rash. Additionally, when a patient transfers between hospitals without returning to the hospital where the patient was first admitted, the record in the data set will be registered as censored from the moment of hospital transfer. This is because a unique hospital patient identification number is created in each hospital, which does not match with previously created records for that patient. Furthermore, general laboratory results are only available for approximately 10% of the patients. The number of patients with mCRPC may be underestimated since the patients identified with nmCRPC were not re-identified as mCRPC when metastasis occurred during the study period. Furthermore, inclusion of lenient criteria for medical castration with the last PSA levels of > 2 ng/mL for patients with mCNPC presents a risk for misclassification of mCNPC as nmCRPC.

Treatment with systemic corticosteroids for various immune-related disorders, could have resulted in an overestimation of the treatment for skin rash. Also, it should be noted that not all ICD-10 codes used to identify allergies and hypersensitivities [26], may be related to allergies and hypersensitivities such as contact dermatitis, urticaria and keratitis.

## Conclusion

The finding from this real-world study in patients with advance PC from Japan demonstrate that, nearly 20–30% patients experience skin rash. This value lies within the range of what has been observed for PC patients in an RWE study of the US [12], and the subgroup analysis of the Japanese SPARTAN and TITAN clinical trial [13]. The PC treatments from the RWE study of the US (bicalutamide, enzalutamide, abiraterone acetate plus prednisolone), are also used by a considerable proportion of patients in our study. More specifically, the total proportion of patients using bicalutamide, enzalutamide, abiraterone acetate ranges from 60 to 85%. Because the incidence of skin rash is considerably lower in the overseas study compared to our study (10.9% vs. 20–29%), the results suggest the Japanese population may be more at risk to develop skin rash. However, it should be noted that the methods to identify skin rash between both studies differ and that this may confound the results. The results of the Cox regression analysis showed a possible causality whereby prior allergy or hypersensitivity event, BMI, higher age and higher baseline PSA levels may be linked to the time to skin rash.

Systemic and topical corticosteroids were the preferred treatment options for the management of skin rash. The median duration of skin rash treatment with topical corticosteroids was relatively shorter compared to systematic corticosteroids and oral antihistamine across all cohorts. The results should be viewed in light of the existing limitations associated with this database analysis.

## Supplementary Information

**Additional file 1.** Supplementary material.

## Data Availability

The data is available upon request by contact details provided in the manuscript.

## Abbreviations

AEs: Adverse events
ALT: Alanine aminotransferase
ALP: Alkaline phosphatase
ADT: Androgen deprivation therapy
AST: Aspartate aminotransferase
BMI: Body mass index
BSA: Body surface area
CI: Confidence interval
eGFR: Estimated glomerular filtration rate
GGT: Gamma-glutamyltransferase
HR: Hazard ratio
IQR: Interquartile range
L: Liter
mCRPC: Metastatic castration-resistant prostate cancer patients
mCNPC: Metastatic castration-naïve prostate cancer patients
MDV: Medical Data Vision
Max: Maximum
min: Minutes
Min: Minimum
N: Number of patients in a skin rash specific group
n: Number of patients without missing value in a specific group
nmCRPC: Non-metastatic castration-resistant prostate cancer
ng: Nanogram
mL: Milliliter
PC: Prostate cancer
PSA: Prostate-specific antigen
Q1: First quartile
Q3: Third quartile
RP: Radical prostatectomy
RT: Radiotherapy
Ref: Reference level
SD: Standard deviation
SGLT2: Sodium-dependent glucose co-transporter type 2
US: United States
U: Units

## References

[1] Bray F, Ferlay J, Soerjomataram I, Siegel RL, Torre LA, Jemal A (2018). Global cancer statistics 2018: GLOBOCAN estimates of incidence and mortality worldwide for 36 cancers in 185 countries. CA Cancer J Clin.

[2] Center MM, Jemal A, Lortet-Tieulent J, Ward E, Ferlay J, Brawley O, Bray F (2012). International variation in prostate cancer incidence and mortality rates. Eur Urol.

[3] Saika T, Miura N, Fukumoto T, Yanagihara Y, Miyauchi Y, Kikugawa T (2018). Role of robot-assisted radical prostatectomy in locally advanced prostate cancer. Int J Urol.

[4] Ito K (2014). Prostate cancer in Asian men. Nat Rev Urol.

[5] Kakehi Y, Sugimoto M, Taoka R (2017). Evidenced-based clinical practice guideline for prostate cancer (summary: Japanese Urological Association, 2016 edition). Int J Urol.

[6] Freedland SJ, Humphreys EB, Mangold LA, Eisenberger M, Dorey FJ, Walsh PC, Partin AW (2007). Death in patients with recurrent prostate cancer after radical prostatectomy: prostate-specific antigen doubling time subgroups and their associated contributions to all-cause mortality. J Clin Oncol.

[7] Hussain M, Fizazi K, Saad F, Rathenborg P, Shore N, Ferreira U, Ivashchenko P, Demirhan E, Modelska K, Phung D (2018). Enzalutamide in men with nonmetastatic, castration-resistant prostate cancer. N Engl J Med.

[8] Smith MR, Saad F, Chowdhury S, Oudard S, Hadaschik BA, Graff JN, Olmos D, Mainwaring PN, Lee JY, Uemura H (2018). Apalutamide treatment and metastasis-free survival in prostate cancer. N Engl J Med.

[9] Sumanasuriya S, De Bono J (2018). Treatment of advanced prostate cancer: a review of current therapies and future promise. Cold Spring Harb Perspect Med.

[10] Rice MA, Malhotra SV, Stoyanova T (2019). Second-generation antiandrogens: from discovery to standard of care in castration resistant prostate cancer. Front Oncol.

[11] Nguyen PL, Alibhai SM, Basaria S, D'Amico AV, Kantoff PW, Keating NL, Penson DF, Rosario DJ, Tombal B, Smith MR (2015). Adverse effects of androgen deprivation therapy and strategies to mitigate them. Eur Urol.

[12] Shah A, Shah R, Kebede N, Mohamed A, Botteman M, Waldeck R, Hussain A (2020). Real-world incidence and burden of adverse events among non-metastatic prostate cancer patients treated with secondary hormonal therapies following androgen deprivation therapy. J Med Econ.

[13] Uemura H, Koroki Y, Iwaki Y, Imanaka K, Kambara T, Lopez-Gitlitz A, Smith A, Uemura H (2020). Skin rash following administration of apalutamide in Japanese patients with Advanced Prostate Cancer: an integrated analysis of the phase 3 SPARTAN and TITAN studies and a phase 1 open-label study. BMC Urol.

[14] Ikoma A, Ebata T, Fukuda R, Takase Y, Taniguchi N, Takemura K, Vaglio J, Poncet M, LeClercq D (2020). Prevalence of pruritus in the elderly with dementia: a multicenter survey of Japanese patients. Acta Derm Venereol.

[15] Kimura N, Nakagami G, Takehara K, Miura Y, Nakamura T, Kawashima M, Tsunemi Y, Sanada H (2013). Prevalence of asteatosis and asteatotic eczema among elderly residents in facilities covered by long-term care insurance. J Dermatol.

[16] Blume-Peytavi U, Kottner J, Sterry W, Hodin MW, Griffiths TW, Watson RE, Hay RJ, Griffiths CE (2016). Age-associated skin conditions and diseases: current perspectives and future options. Gerontologist.

[17] Iguchi T, Kimura G, Fukasawa S, Suzuki H, Uemura H, Nishimura K, Matsumoto H, Yokomizo A, Armstrong AJ, Rosbrook B (2021). Enzalutamide with androgen deprivation therapy in Japanese men with metastatic hormone-sensitive prostate cancer: a subgroup analysis of the phase III ARCHES study. Int J Urol.

[18] Tinsley SM, Kurtin SE, Ridgeway JA (2015). Practical management of lenalidomide-related rash. Clin Lymphoma Myeloma Leuk.

[19] Sakaeda T, Kobuchi S, Yoshioka R, Haruna M, Takahata N, Ito Y, Sugano A, Fukuzawa K, Hayase T, Hayakawa T (2018). Susceptibility to serious skin and subcutaneous tissue disorders and skin tissue distribution of sodium-dependent glucose co-transporter type 2 (SGLT2) inhibitors. Int J Med Sci.

[20] Tohi Y, Kato T, Fukuhara H, Kobayashi K, Ohira S, Ikeda K, Daizumoto K, Katayama S, Shimizu R, Nishimura K et al. Real-world analysis of apalutamide-associated skin adverse events in Japanese patients with advanced prostate cancer: a multi-institutional study in the Chu-shikoku Japan Urological Consortium. Int J Clin Oncol (2022) 10.1007/s10147-022-02183-z 35596089

[21] About MDV Database. https://en.mdv.co.jp/about-mdv-database/. Accessed 23 Aug 2022.

[22] ICD-10. International statistical classification of diseases and related health problems. 5th ed. World Health Organization; 2016

[23] Borley N, Feneley MR (2009). Prostate cancer: diagnosis and staging. Asian J Androl.

[24] European Medicines Agency (EMA). Guideline on the evaluation of anticancer medicinal products in man. https://www.ema.europa.eu/en/evaluation-anticancer-medicinal-products-man. Accessed 24 Nov 2021.

[25] Ji C, Guha M, Zhu X, Whritenour J, Hemkens M, Tse S, Walker GS, Evans E, Khan NK, Finkelstein MB, Callegari E, Obach RS (2020). Enzalutamide and apalutamide: in vitro chemical reactivity studies and activity in a mouse drug allergy model. Chem Res Toxicol.

[26] Tanno LK, Calderon M, Demoly P (2016). Joint Allergy Academies. Supporting the validation of the new allergic and hypersensitivity conditions section of the World Health Organization International Classification of Diseases-11. Asia Pac Allergy.

[27] Deo SV, Deo V, Sundaram V (2021). Survival analysis-part 2: Cox proportional hazards model. Indian J Thorac Cardiovasc Surg.

[28] Wang L, Lu B, He M, Wang Y, Wang Z, Du L (2022). Prostate cancer incidence and mortality: global status and temporal trends in 89 countries from 2000 to 2019. Front Public Health.

[29] U.S. Food & Drug Administration. https://www.accessdata.fda.gov/drugsatfda_docs/label/2018/203415s014lbl.pdf. Accessed 23 Sep 2022.

[30] Chi KN, Agarwal N, Bjartell A, Chung BH, Pereira de Santana Gomes AJ, Given R, Juárez Soto Á, Merseburger AS, Özgüroğlu M, Uemura H (2019). Apalutamide for metastatic, castration-sensitive prostate cancer. N Engl J Med.

[31] Kobayashi T, Kinoshita H, Nishizawa K, Mitsumori K, Ogawa O, Kamoto T (2005). Age-associated increase of prostate-specific antigen in a high level of men visiting urological clinics. Int J Urol.

