# Supplementary material for: A retrospective study on the incidence, management and risk factors of skin rash in patients with advanced prostate cancer in Japan
Source: BMC Urol. 2023 Apr 28;23:73. doi: 10.1186/s12894-023-01246-1 (PMC10148545; doi:10.1186/s12894-023-01246-1)
Supplement: Supplementary file 1 — Additional file 1. Supplementary material. [file 12894_2023_1246_MOESM1_ESM.docx]

**A retrospective study on the incidence, management and risk factors of skin rash in patients**

**with advanced prostate cancer in Japan**

Raf De Moor^1*^, Yosuke Koroki^2^, David Bin-Chia Wu^3,4,5^, Dae Young Yu^3^, Mikiko Tohyama^6^, Chikara Ohyama^7^

^1^ Value, Evidence and Access Department, Janssen Pharmaceutical KK, Tokyo, Japan

^2^ Medical Affairs, Janssen Pharmaceutical KK, Tokyo, Japan

^3^ Janssen Pharmaceutical Companies of Johnson and Johnson, Asia Pacific Regional Office, Singapore

^4^ Saw Swee Hock School of Public Health, National University of Singapore, Singapore

^5^ School of Pharmacy, Monash University Malaysia, Malaysia

^6^ Department of Dermatology, National Hospital Organization Shikoku Cancer Center, Matsuyama, Japan

^7^Department of Urology, Hirosaki University Graduate School of Medicine, Hirosaki, Japan

***^*^***Corresponding author

Raf De Moor

Janssen Pharmaceutical KK

3 Chome-5-2 Nishikanda, Chiyoda City

Tokyo 101-0065

rdemoor.@its.jnj.com

**SUPPLEMENTARY MATERIAL**

**Identification skin rash**

# Table S1. ICD-10 skin rash codes

| **ICD-10 code** | **Description** |
| --- | --- |
| L10 | Pemphigus |
| L11 | Other acantholytic disorders |
| L12 | Pemphigoid |
| L13 | Other bullous disorders |
| L14 | Bullous disorders in diseases classified elsewhere |
| L20 | Atopic dermatitis |
| L21 | Seborrheic dermatitis |
| L22 | Diaper dermatitis |
| L23 | Allergic contact dermatitis |
| L24 | Irritant contact dermatitis |
| L25 | Unspecified contact dermatitis |
| L26 | Exfoliative dermatitis |
| L27 | Dermatitis due to substances taken internally |
| L28 | Lichen simplex chronicus and prurigo |
| L29 | Pruritus |
| L30 | Other and unspecified dermatitis |
| L40 | Psoriasis |
| L41 | Parapsoriasis |
| L42 | Pityriasis rosea |
| L43 | Lichen planus |
| L44 | Other papulosquamous disorders |
| L45 | Papulosquamous disorders in diseases classified elsewhere |
| L50 | Urticaria |
| L51 | Erythema multiforme |
| L52 | Erythema nodosum |
| L53 | Other erythematous conditions |
| L54 | Erythema in diseases classified elsewhere |
| L60 | Nail disorders |
| L62 | Nail disorders in diseases classified elsewhere |
| L63 | Alopecia areata |
| L64 | Androgenic alopecia |
| L65 | Other nonscarring hair loss |
| L66 | Cicatricial alopecia [scarring hair loss] |
| L67 | Hair color and hair shaft abnormalities |
| L68 | Hypertrichosis |
| L70 | Acne |
| L71 | Rosacea |
| L72 | Follicular cysts of skin and subcutaneous tissue |
| L73 | Other follicular disorders |
| L74 | Eccrine sweat disorders |
| L75 | Apocrine sweat disorders |
| L80 | Vitiligo |
| L81 | Other disorders of pigmentation |
| L82 | Seborrheic keratosis |
| L83 | Acanthosis nigricans |
| L84 | Corns and callosities |
| L85 | Other epidermal thickening |
| L86 | Keratoderma in diseases classified elsewhere |
| L87 | Transepidermal elimination disorders |
| L88 | Pyoderma gangrenosum |
| L89 | Pressure ulcer |
| L90 | Atrophic disorders of skin |
| L91 | Hypertrophic disorders of skin |
| L92 | Granulomatous disorders of skin and subcutaneous tissue |
| L93 | Lupus erythematosus |
| L94 | Other localized connective tissue disorders |
| L95 | Vasculitis limited to skin, not elsewhere classified |
| L97 | Non-pressure chronic ulcer of lower limb, not elsewhere classified |
| L98 | Other disorders of skin and subcutaneous tissue, not elsewhere classified |
| L99 | Other disorders of skin and subcutaneous tissue in diseases classified elsewhere |
| R21 | Rash and other nonspecific skin eruption |
| R23 | Other skin changes |

# Table S2. ICD-10 skin rash treatments

| **Atccode** | **Drug name** |
| --- | --- |
| H02B0 | Betamethasone/d-Chlorpheniramine Maleate |
| R06A0 | d-Chlorpheniramine Maleate |
| R06A0 | Chlorpheniramine Maleate |
| R06A0 | Alimemazine Tartrate |
| R06A0 | Ebastine |
| R06A0 | Epinastine Hydrochloride |
| R06A0 | Emedastine Fumarate |
| R06A0 | Oxatomide |
| R06A0 | Olopatadine Hydrochloride |
| R06A0 | Clemastine Fumarate |
| R06A0 | Chlorpheniramine Maleate |
| R06A0 | Diphenhydramine Hydrochloride |
| R06A0 | Cyproheptadine Hydrochloride Hydrate |
| R06A0 | Cetirizine Hydrochloride |
| R06A0 | Diphenhydramine Tannate |
| R06A0 | Desloratadine |
| R06A0 | Promethazine Hibenzate |
| R06A0 | Bilastine |
| R06A0 | Fexofenadine Hydrochloride |
| R06A0 | Promethazine Methylenedisalicylate |
| R06A0 | Promethazine Hydrochloride |
| R06A0 | Bepotastine Besilate |
| R06A0 | Homochlorcyclizine Hydrochloride |
| R06A0 | Mequitazine |
| R06A0 | Rupatadine Fumarate |
| R06A0 | Levocetirizine Hydrochloride |
| R06A0 | Loratadine |
| H02A1 | Dexamethasone Palmitate |
| H02A1 | Dexamethasone Sodium Phosphate |
| H02A1 | Triamcinolone Acetonide |
| H02A1 | Hydrocortisone Sodium Succinate |
| H02A1 | Hydrocortisone Sodium Phosphate |
| H02A1 | Prednisolone Sodium Succinate |
| H02A1 | Betamethasone Sodium Phosphate |
| H02A1 | Methylprednisolone Sodium Succinate |
| H02A1 | Methylprednisolone Acetate |
| H02A2 | Cortisone Acetate |
| H02A2 | Dexamethasone |
| H02A2 | Triamcinolone |
| H02A2 | Hydrocortisone |
| H02A2 | Fludrocortisone Acetate |
| H02A2 | Prednisolone |
| H02A2 | Betamethasone |
| H02A2 | Methylprednisolone |
| H02B0 | Betamethasone Acetate/Betamethasone Sodium Phosphate |
| D07A0 | Amcinonide |
| D07A0 | Alclometasone Dipropionate |
| D07A0 | Clobetasol Propionate |
| D07A0 | Clobetasone Butyrate |
| D07A0 | Diflucortolone Valerate |
| D07A0 | Difluprednate |
| D07A0 | Diflorasone Diacetate |
| D07A0 | Dexamethasone |
| D07A0 | Dexamethasone Propionate |
| D07A0 | Dexamethasone Valerate |
| D07A0 | Deprodone Propionate |
| D07A0 | Triamcinolone Acetonide |
| D07A0 | Hydrocortisone Butyrate |
| D07A0 | Fluocinonide |
| D07A0 | Fluocinolone Acetonide |
| D07A0 | Fludroxycortide |
| D07A0 | Prednisolone |
| D07A0 | Prednisolone Farnesylate |
| D07A0 | Prednisolone Valerate Acetate |
| D07A0 | Beclometasone Dipropionate |
| D07A0 | Betamethasone Dipropionate |
| D07A0 | Betamethasone Valerate |
| D07A0 | Betamethasone Butyrate Propionate |
| D07A0 | Mometasone Furoate |
| D07A0 | Hydrocortisone Butyrate Propionate |
| D07B1 | Oxytetracycline Hydrochloride/Hydrocortisone |
| D07B1 | Chloramphenicol/Fradiomycin Sulfate/Prednisolone Combination |
| D07B1 | Fradiomycin Sulfate/Prednisolone |
| D07B1 | Fluocinolone Acetonide/Fradiomycin Sulfate |
| D07B1 | Betamethasone Valerate/Gentamicin Sulfate |
| D07B1 | Betamethasone Valerate/Fradiomycin Sulfate |
| D07B1 | Mixed Killed Bacteria/Hydrocortisone |
| D07B4 | Hydrocortisone/Crotamiton |
| D07B4 | Hydrocortisone Acetate/Fradiomycin Combination |

# Table S3. ICD-10 eosinophilia codes

| ICD-10 code | Description |
| --- | --- |
| D72.1 | Eosinophilia |
| D72.10 | Eosinophilia, unspecified |
| D72.11 | Hypereosinophilic syndrome |
| D72.110 | Idiopathic hypereosinophilic syndrome |
| D72.111 | Lymphocytic Variant Hypereosinophilic Syndrome |
| D72.118 | Other hypereosinophilic syndrome |
| D72.119 | Hypereosinophilic syndrome, unspecified |
| D72.12 | Drug rash with eosinophilia and systemic symptoms syndrome |
| D72.18 | Eosinophilia in diseases classified elsewhere |
| D72.19 | Other eosinophilia |

# Table S4. ICD-10 codes allergy and hypersensitivity

| ICD-10 code | Description |
| --- | --- |
| D69 | Purpura and other haemorrhagic conditions |
| D69.0 | Allergic purpura |
| H10 | Conjunctivitis |
| H10.1 | Acute atopic conjunctivitis |
| H10.4 | Chronic conjunctivitis |
| H10.5 | Blepharoconjunctivitis |
| H16 | Keratitis |
| H16.2 | Keratoconjunctivitis |
| H18 | Other disorders of cornea |
| H18.6 | Keratoconus |
| J30 | Vasomotor and allergic rhinitis |
| J30.0 | Vasomotor rhinitis |
| J30.1 | Allergic rhinitis due to pollen |
| J30.2 | Other seasonal allergic rhinitis |
| J30.3 | Other allergic rhinitis |
| J30.4 | Allergic rhinitis, unspecified |
| J32 | Chronic sinusitis |
| J33 | Nasal polyp |
| J45 | Asthma |
| J45.0 | Predominantly allergic asthma |
| J45.1 | Nonallergic asthma |
| J45.8 | Mixed asthma |
| J45.9 | Asthma, unspecified |
| J46 | Status asthmaticus |
| J67 | Hypersensitivity pneumonitis due to organic dust |
| J67.0 | Farmer lung |
| J67.1 | Bagassosis |
| J67.2 | Bird fancier lung |
| J67.3 | Suberosis |
| J67.4 | Maltworker lung |
| J67.5 | Mushroom-worker lung |
| J67.6 | Maple-bark-stripper lung |
| J67.7 | Air-conditioner and humidifier lung |
| J67.8 | Hypersensitivity pneumonitis due to other organic dusts |
| J67.9 | Hypersensitivity pneumonitis due to unspecified organic dust |
| K20 | Oesophagitis |
| K52 | Other noninfective gastroenteritis and colitis |
| K52.2 | Allergic and dietetic gastroenteritis and colitis |
| K52.8 | Other specified noninfective gastroenteritis and colitis (Incl.: Eosinophilic gastritis or gastroenteritis) |
| L20 | Atopic dermatitis |
| L20.0 | Besnier prurigo |
| L20.8 | Other atopic dermatitis |
| L20.9 | Atopic dermatitis, unspecified |
| L23 | Allergic contact dermatitis |
| L23.0 | Allergic contact dermatitis due to metals |
| L23.1 | Allergic contact dermatitis due to adhesives |
| L23.2 | Allergic contact dermatitis due to cosmetics |
| L23.3 | Allergic contact dermatitis due to drugs in contact with skin |
| L23.4 | Allergic contact dermatitis due to dyes |
| L23.5 | Allergic contact dermatitis due to other chemical products |
| L23.6 | Allergic contact dermatitis due to food in contact with skin |
| L23.7 | Allergic contact dermatitis due to plants, except food |
| L23.8 | Allergic contact dermatitis due to other agents |
| L23.9 | Allergic contact dermatitis, unspecified cause |
| L27 | Dermatitis due to substances taken internally L27.0 Generalized skin eruption due to drugs and medicaments |
| L27.1 | Localized skin eruption due to drugs and medicaments |
| L27.2 | Dermatitis due to ingested food |
| L27.8 | Dermatitis due to other substances taken internally |
| L27.9 | Dermatitis due to unspecified substance taken internally |
| L50 | Urticaria |
| L50.0 | Allergic urticaria |
| L50.1 | Idiopathic urticaria |
| L50.2 | Urticaria due to cold and heat |
| L50.3 | Dermatographic urticaria |
| L50.4 | Vibratory urticaria |
| L50.5 | Cholinergic urticaria |
| L50.6 | Contact urticaria |
| L50.8 | Other urticaria |
| L50.9 | Urticaria, unspecified |
| L51 | Erythema multiforme |
| L51.0 | Nonbullous erythema multiforme |
| L51.1 | Bullous erythema multiforme |
| L51.2 | Toxic epidermal necrolysis [Lyell] |
| L51.8 | Other erythema multiforme |
| L51.9 | Erythema multiforme, unspecified |
| L52 | Erythema nodosum |
| T78 | Adverse effects, not elsewhere classified T78.0 Anaphylactic shock due to adverse food reaction |
| T78.1 | Other adverse food reactions, not elsewhere classified |
| T78.2 | Anaphylactic shock, unspecified |
| T78.3 | Angioneurotic oedema |
| T78.4 | Allergy, unspecified |
| T78.8 | Other adverse effects, not elsewhere classified |
| T78.9 | Adverse effect, unspecified |
| T80 | Complications following infusion, transfusion and therapeutic injection |
| T80.5 | Anaphylactic shock due to serum |
| Z01.5 | Diagnostic skin and sensitization tests |
| Z88.0 | Personal history of allergy to penicillin |
| Z88.1 | Personal history of allergy to other antibiotic agents |
| Z88.2 | Personal history of allergy to sulfonamides |
| Z88.3 | Personal history of allergy to other anti-infective agents |
| Z88.4 | Personal history of allergy to anaesthetic agent |
| Z88.5 | Personal history of allergy to narcotic agent |
| Z88.6 | Personal history of allergy to analgesic agent |
| Z88.7 | Personal history of allergy to serum and vaccine |
| Z88.8 | Personal history of allergy to other drugs, medicaments and biological substances |
| Z88.9 | Personal history of allergy to unspecified drugs, medicaments and biological substances |
| Z91.0 | Personal history of allergy, other than to drugs and biological substances |

**Sensitivity Result Tables**

# Table S5. Patient characteristics (Sensitivity)

|  | **Overall** | **nmCRPC** | **mCRPC** | | **mCNPC** | |
| --- | --- | --- | --- | --- | --- | --- |
|  | **N = 2,422** | **N = 1,626** | **N = 539** | | **N = 428** | |
| **Age on the index date (years)** | | | | | | |
| Mean (SD) | 77.5 (8.05) | 78.3 (7.98) | 76.3 (7.88) | | 76.0 (8.35) | |
| Median | 78 (46:98) | 79 (51:98) | 76 (46:97) | | 76(50:96) | |
| IQR | 11 (72:83) | 11 (73:84) | 11 (71:82) | | 12 (70:82) | |
| **Age group at the index date, n (%)** | | |  |  |  |  |
| 18 ‑ 49 | 1 (0.04%) | 0 (0.00%) | 1 (0.19%) | | 0 (0.00%) | |
| 50 ‑ 59 | 35 (1.45%) | 20 (1.23%) | 9 (1.67%) | | 10 (2.34%) | |
| 60 ‑ 69 | 372 (15.36%) | 223 (13.71%) | 90 (16.70%) | | 84 (19.63%) | |
| 70 ‑ 79 | 975 (40.26%) | 620 (38.13%) | 248 (46.01%) | | 177 (41.36%) | |
| 80 ‑ 89 | 908 (37.49%) | 661 (40.65%) | 170 (31.54%) | | 137 (32.01%) | |
| 90+ | 131 (5.41%) | 102 (6.27%) | 21 (3.90%) | | 20 (4.67%) | |
| Total | 2,422 (100%) | 1,626 (100%) | 539 (100%) | | 428 (100%) | |
| **Previous treatment for prostate cancer, n (%)** | | | | | | |
| Any radical Prostatectomy (RP) | 58 (2.39%) | 42 (2.58%) | 11 (2.04%) | | 6 (1.40%) | |
| Any radiotherapy (RT) | 175 (7.23%) | 102 (6.27%) | 64 (11.87%) | | 26 (6.07%) | |
| Any hormonal therapy | 1,779 (73.45%) | 1,289 (79.27%) | 465 (86.27%) | | 145 (33.88%) | |
| **Baseline PSA index date (ng/mL)** |  |  |  | |  | |
| n | 2,269 | 1,626 | 539 | | 275 | |
| Mean | 114.40 (618.24) | 25.79 (228.66) | 144.56 (571.15) | | 644.43 (1,454.11) | |
| Median | 5.48 (0: 12,676.31) | 4.43 (2.006: 8,519.69) | 12.60 (2.01; 9,070.83) | | 103.35(0: 12,676.31) | |
| IQR | 15.66  (2.962: 18.62) | 6.74  (2.8: 9.54) | 54.09  (4.439: 58.52) | | 589.34  (17.36: 606.70) | |

N ‑ number of patients in a specific group, n ‑ number of patients without missing value in a specific group.

IQR ‑ Interquartile range; Max ‑ Maximum; mCNPC ‑ Metastatic castration‑naïve prostate cancer; mCRPC ‑ Metastatic castration‑resistant prostate cancer; Min ‑ Minimum; nmCRPC ‑ Non‑metastatic castration‑resistant prostate cancer; PSA ‑ Prostate‑specific antigen; RP ‑ Radical prostatectomy; RT ‑ Radiotherapy; SD ‑ Standard deviation.

# Table S6. Incidence and treatment duration of skin rash, and drugs used for prostate cancer (Sensitivity)

|  | **nmCRPC** | **mCRPC** | **mCNPC** |
| --- | --- | --- | --- |
|  | **N = 1,626** | **N = 539** | **N = 428** |
| **Incidence proportion of skin rash (n, %)^1^** | | | |
| Number of patients with skin rash | 274 (16.85%) | 120 (22.26%) | 116 (27.10%) |
| **Incidence rate of skin rash (per patient year)^1^** | | | |
| Patient years at risk (patient years) | 3,450.80 | 806.41 | 781.27 |
| Incidence rate (per 100 patient years) | 7.94 | 14.88 | 14.85 |
| 95% CI (lower ‑ upper) | 7.05-8.94 | 12.44-17.80 | 12.38-17.81 |
| **Duration of skin rash (days)** | | | |
| n | 274 | 120 | 116 |
| Mean (SD) | 133.90 (234.56) | 112.21 (183.72) | 108.55 (179.28) |
| Median | 35.5 (1: 1401) | 34 (1: 1196) | 28 (1: 1023) |
| IQR | 92 (28:120) | 79.5 (28: 107.5) | 64.5 (28:92.5) |
| **Drugs used for treatment of prostate cancer between index date (inclusive) and the first onset of skin rash (exclusive) (n, %)^2^** | | | |
| n | 274 | 120 | 116 |
| Goserelin | 95 (34.67%) | 32 (26.67%) | 20 (17.24%) |
| leuprorelin acetate | 132 (48.18%) | 47 (39.17%) | 36 (31.03%) |
| Degarelix | 23 (8.39%) | 29 (24.17%) | 39 (33.62%) |
| Flutamide | 65 (23.72%) | 28 (23.33%) | 20 (17.24%) |
| Bicalutamide | 118 (43.07%) | 37 (30.83%) | 77 (66.38%) |
| Enzalutamide | 65(23.72%) | 27 (22.50%) | 10 (8.62%) |
| Apalutamide | 11 (4.01%) | 2 (1.67%) | 2 (1.72%) |
| Abiraterone Acetate (plus prednisolone) | 32 (11.68%) | 22 (18.33%) | 16 (13.79%) |
| Darolutamide | 0 (0.00%) | 1 (0.83%) | 0 (0.00%) |
| Docetaxel | 59 (21.53%) | 44 (36.67%) | 15 (12.93%) |
| Cabazitaxel | 13 (4.74%) | 11 (9.17%) | 3 (2.59%) |
| radium‑223 | 0 (0.00%) | 5 (4.17%) | 1 (0.86%) |
| Pembrolizumab | 0 (0.00%) | 1 (0.83%) | 0 (0.00%) |

^1^Estimated by exact Poisson confidence interval.

^2^Percentage among the number of patients with skin rash.

N ‑ Number of patients in a specific group, n ‑ Number of patients occurred skin rash in a specific group.

CI ‑ Confidence interval; IQR ‑ Interquartile range; Max ‑ Maximum; mCNPC ‑ Metastatic castration‑naïve prostate cancer; mCRPC ‑ Metastatic castration‑resistant prostate cancer; Min ‑ Minimum; nmCRPC - Non‑metastatic castration‑resistant prostate cancer; SD ‑ Standard deviation

# Table S7. Cox regression for time to skin rash (Sensitivity)

|  | **nmCRPC** | | | | **mCRPC** | | | | **mCNPC** | | | |
| --- | --- | --- | --- | --- | --- | --- | --- | --- | --- | --- | --- | --- |
|  | **HR** | **95% CI  (Lower)** | **95% CI  (Upper)** | **p‑value** | **HR** | **95% CI  (Lower)** | **95% CI  (Upper)** | **p‑value** | **HR** | **95% CI  (Lower)** | **95% CI  (Upper)** | **p‑value** |
| **Age group at the index date** | | | | | | | | | | | | |
| 18 ‑ 49 | ‑ |  |  |  | 1.40 | 0.17 | 11.76 | 0.7595 | ‑ |  |  |  |
| 50 ‑ 59 | 1 |  |  | Ref | 1 |  |  | Ref | 1 |  |  | Ref |
| 60 ‑ 69 | 1.99 | 1.14 | 3.47 | 0.0159 | 1.11 | 0.47 | 2.63 | 0.8178 | 0.95 | 0.40 | 2.22 | 0.9004 |
| 70 ‑ 79 | 2.33 | 1.35 | 4.02 | 0.0025 | 1.49 | 0.64 | 3.45 | 0.3581 | 0.94 | 0.41 | 2.14 | 0.8771 |
| 80 ‑ 89 | 3.19 | 1.84 | 5.51 | <0.0001 | 1.82 | 0.78 | 4.25 | 0.1630 | 1.34 | 0.59 | 3.07 | 0.4873 |
| 90+ | 4.18 | 2.34 | 7.48 | <0.0001 | 3.69 | 1.42 | 9.57 | 0.0072 | 2.33 | 0.93 | 5.88 | 0.0719 |

CI - Confidence interval; HR - Hazard ratio; **mCNPC** ‑ Metastatic castration‑naïve prostate cancer; mCRPC ‑ Metastatic castration‑resistant prostate cancer; nmCRPC ‑ Non‑metastatic castration‑resistant prostate cancer; PSA - Prostate-specific antigen; Ref - Reference level; RP ‑ Radical prostatectomy; RT - Radiotherapy.

# Table S8. Summary of prescribed medicines for management of skin rash (Sensitivity)

|  | **nmCRPC** | | **mCRPC** | | **mCNPC** | |
| --- | --- | --- | --- | --- | --- | --- |
|  | **N = 274** | | **N = 120** | | **N = 116** | |
| **Types of skin rash treatment, n (%)** | | | | | | |
| Oral antihistamine^1^ | 79 (28.83%) | | 28 (23.33%) | | 36 (31.03%) | |
| Systemic corticosteroid^1^ | 134 (48.91%) | | 77 (64.17%) | | 57 (49.14%) | |
| Topical corticosteroid | 163 (59.49%) | | 54 (45.00%) | | 65 (56.03%) | |
| **Duration of skin rash treatment** | | | | | | |
| Oral antihistamine^1^ |  |  |  |  |  |  |
| Number of patients using the treatment | 79 | | 28 | | 36 | |
| Mean | 88.44 (193.52) | | 58.71 (84.89) | | 140.67 (214.92) | |
| Median | 22 (1:1169) | | 25 (3:324) | | 37 (1:872) | |
| IQR | 79 (7:86) | | 37.5 (13:50.5) | | 155 (13:168) | |
| Systemic corticosteroid^1^ |  |  |  |  |  |  |
| Number of patients using the treatment | 134 | | 77 | | 57 | |
| Mean | 166.94 (256.21) | | 114.31 (164.10) | | 127.02 (189.75) | |
| Median | 63 (1:1430) | | 58 (1:753) | | 46 (1:1022) | |
| IQR | 161 (21:182) | | 107 (21:128) | | 133 (18:151) | |
| Topical corticosteroid |  |  |  |  |  |  |
| Number of patients using the treatment | 163 * | | 54 | | 65 | |
| Mean | 79.33 (164.45) | | 92.30 (183.19) | | 73.15 (149.14) | |
| Median | 28 (28:1350) | | 28 (28:1223) | | 28 (28:981) | |
| IQR (Q1:Q3) | 28 (28:56) | | 56 (28:84) | | 4 (28:32) | |

^1^Including combination of systemic antihistamine and corticosteroid.

N ‑ Number of patients in rash specific group, n ‑ Number of patients without missing value in a specific group.

IQR ‑ Interquartile range; Max - Maximum; **mCNPC** ‑ Metastatic castration‑naïve prostate cancer; mCRPC ‑ Metastatic castration‑resistant prostate cancer; Min - Minimum; nmCRPC ‑ Non‑metastatic castration‑resistant prostate cancer; SD - Standard deviation.

# Table S9. Pearson’s correlation method

|  | **Coefficient** | **95% CI (lower)** | **95% CI (upper)** | **p value** |
| --- | --- | --- | --- | --- |
| **mCRPC** | 0.07 | -0.0044 | 0.1513 | 0.06435 |
| **nmCRPC** | 0.01 | -0.0384 | 0.0545 | 0.7339 |
| **mCNPC** | 0.04 | -0.1636 | 0.0764 | 0.4722 |
